# Imaging Single Particle Profiler to Study Nanoscale Bioparticles Using Conventional Confocal Microscopy

**DOI:** 10.1021/acs.nanolett.4c05117

**Published:** 2025-01-29

**Authors:** Taras Sych, André Görgens, Loïc Steiner, Gozde Gucluler, Ylva Huge, Farhood Alamdari, Markus Johansson, Firas Aljabery, Amir Sherif, Susanne Gabrielsson, Samir El Andaloussi, Erdinc Sezgin

**Affiliations:** † Science for Life Laboratory, Department of Women’s and Children’s Health, Karolinska Institutet, Tomtebodavägen 23, 17165 Solna, Sweden; ‡ Division of Biomolecular and Cellular Medicine, Department of Laboratory Medicine, Karolinska ATMP Center, Karolinska Institutet, 14152 Huddinge, Sweden; § Department of Cellular Therapy and Allogeneic Stem Cell Transplantation (CAST), Karolinska University Hospital, 14152 Huddinge, Sweden; ∥ Division of Immunology and Respiratory Medicine, Department of Medicine Solna, Karolinska Institutet, 17164 405 Stockholm, Sweden; ⊥ Department of Clinical Immunology and Transfusion Medicine, Center for Molecular Medicine, Karolinska University Hospital, 17164 Stockholm, Sweden; # Department of Urology in Östergötland and Department of Biomedical and Clinical Sciences, Linköping University, 58225 Linköping, Sweden; ∇ Department of Urology, Vastmanland Hospital, 72189 Västerås, Sweden; ○ Departement of Surgery and Urology, County Hospital of Sundsvall-Härnösand, 85643 Sundsvall, Sweden; ◆ Department of Urology in Östergötland, and Department of Biomedical and Clinical Sciences, Linköping University, 58225 Linköping, Sweden; ⬠ Department of Diagnostics and Intervention, Umeå University, 90187 Umeå, Sweden

**Keywords:** single particle profiling, confocal microscopy, liposomes, environmental sensitive probes, membrane fluidity, exosomes, extracellular vesicles

## Abstract

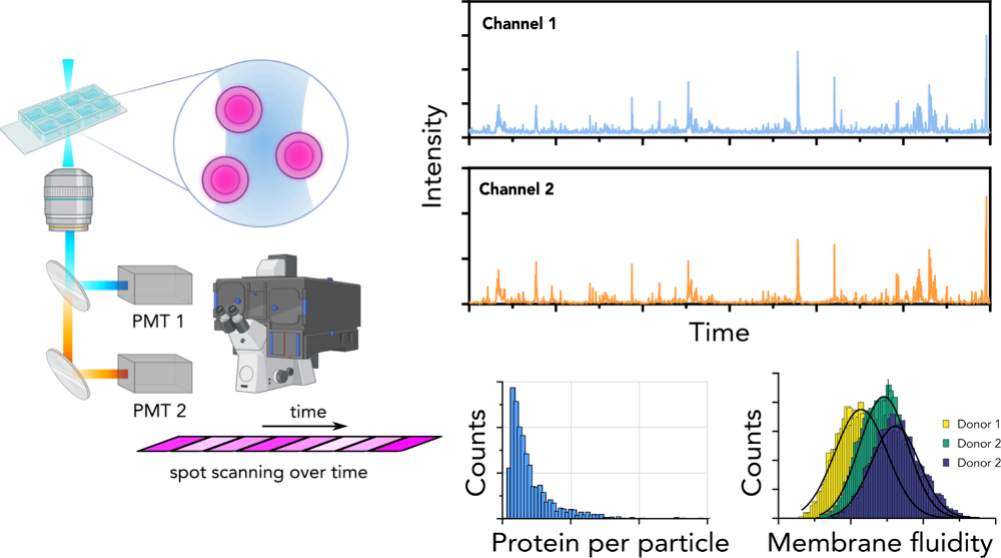

Single particle profiling (SPP) is a unique methodology to study nanoscale bioparticles such as liposomes, lipid nanoparticles, extracellular vesicles, and lipoproteins in a single particle and high throughput manner. The initial version requires the single photon counting modules for data acquisition, which limits its adoptability. Here, we present imaging-based SPP (iSPP) that can be performed by imaging a spot over time in the common imaging mode with confocal detectors. We also provide user-friendly software with a graphical user interface to analyze such data and give quantitative insights on the content and properties of nanoscale bioparticles. We use iSPP to decipher lipid–protein interactions, membrane modifications by drugs, and the heterogeneity of extracellular vesicles isolated from cell lines and human urine. This easily applicable modality of the single particle profiler will facilitate nanoscale bioparticle research in laboratories with access to any confocal microscope.

High throughput screening of biological particles has wide applications in basic and translational research. For cells, flow cytometry is a widely used technique that allows for analyzing tens of thousands of individual cells within minutes, providing information on their surface properties and content. Other bioparticles such as lipoproteins^1^ (LPs) and extracellular vesicles^2^ (EVs) are also commonly used in biomedical research as biomarkers. Quantities of distinct LP types are regularly checked in routine medical examinations to diagnose and prevent metabolic and cardiovascular diseases. Furthermore, it is known that EVs can contain surface proteins designated as disease biomarkers.^3,4^ High throughput screening of such biological particles in a single particle manner is challenging due to their large heterogeneity and small size (10–200 nm). There are a few commercially available instruments for this purpose on the market;^5−7^ however, they are costly and limited in acquisition modalities and analysis. Moreover, their performance decreases as the particle size decreases. We recently developed a high throughput technique, called single particle profiling (SPP), for screening nanometer-sized particles based on widely available confocal microscopy.^8^ We used it for studying artificial liposomes and lipid nanoparticles as well as bioparticles obtained from biological material: lipoproteins, extracellular vesicles, and virus-like particles. SPP requires single photon counting detectors that are not typically included in the base models of confocal microscopes.^9−11^ This limits the adoptability of this new technology.

Here, we introduce imaging SPP (iSPP), single particle profiling performed using the “imaging mode” of standard confocal microscopy, which will increase accessibility of this method and reduce the cost of such measurements. Moreover, we provide here open access software with a graphical user interface that analyzes such data to obtain the content and properties of nanoscale bioparticles. We evaluate the performance of iSPP by addressing several biological questions on nanoscale bioparticles related to protein–lipid interactions, dynamics of drug effect on membranes, and the heterogeneity of biological EVs.

The data acquisition for SPP relies on the ability to record photons emitted by fluorescently labeled particles, moving through the observation volume over time. Such a photon counting setup is often used for fluorescence correlation spectroscopy (FCS); hence, the FCS module was initially employed for data acquisition in SPP.^8^ This requirement for specific photon counting detectors and related software adaptation can be a limiting factor since many confocal microscopes designed for imaging (but not for FCS) do not have such detection and acquisition. The FCS module, however, is not the only mode of a confocal microscope that can yield data as a “fluorescent signal over time”. An image acquired with laser scanning confocal microscopy is a collection of single spots arranged in a grid. The number of such spots is defined by the size of the image and the spatiotemporal resolution of the microscope. Often, to increase the temporal resolution, the dimensions of the images can be reduced. For example, imaging can be performed in only one plane (no *z*-axis) or even in a single line (only *x*-axis) by scanning the same line in one direction over time, which for example, can be used for diffusion measurements in cellular membranes.^12^ Finally, to record a fluorescent signal in one spot over time, there is a mode of “spot scan”. For this, the objective is parked in one position and fluorescence intensity is recorded over time (Figure 1a). We utilized this spot imaging for SPP measurements.

**Figure 1:**
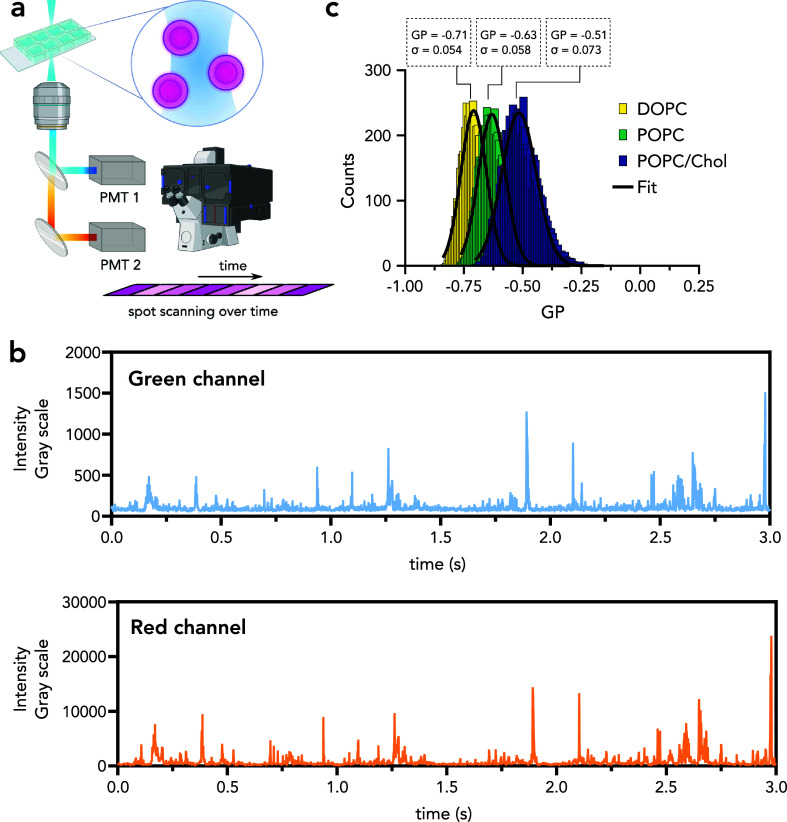
Spot-SPP principle. (a) The objective is fixed on a spot, and GaAsP detectors in integration mode record the fluorescence signal over time in multiple channels in imaging mode. Particles are diffusing through the observation spot; hence, the signal provides intensity fluctuations over time. (b) Fluorescence fluctuations recorded over time in two channels: green channel (490–561 nm) and red channel (650–700 nm). (c) Synthetic liposomes with distinct lipid compositions (pure DOPC, pure POPC, and POPC/Chol (70/30)) are labeled with environmentally sensitive probe NR12S, and fluorescence intensities for single particles in “blue-shifted” (490–561 nm) and “red-shifted” (650–700 nm) channels were recorded. The resulting generalized polarization (GP) histograms for different lipid compositions are presented with the mean and standard deviations.

The major difference between the “spot scan” and the “single photon mode” is the use of the imaging mode of the microscope, e.g., integration mode of the detector (Figure 1a) vs the photon counting mode. While measurements with integration mode will make SPP significantly more accessible, there are a few points to consider for reliable data acquisition (link to the detailed video tutorial can be found in the Materials and Methods section in the Supporting Information). One of the major differences between integration and photon-counting modes is the voltage applied in the integration mode. Detector sensitivity can be altered using the voltage gain; hence, it is particularly important to keep gain settings the same for different samples that will be compared quantitatively. The same applies for excitation laser power when intensities are directly compared. In spot scanning, the intensities are recorded in gray scale values. The dynamic range here can be increased by increasing the bitrate of the image. As in any confocal images, it is important to avoid under- and oversaturation by adjusting laser power, offset, and voltage gain of the detector.

Spot iSPP analyzes the fluorescence fluctuation recorded using a spot scan, i.e., one spot over time. The feasibility of this approach was evaluated using synthetic liposomes labeled with environmentally sensitive dye NR12S.^13^ The emission wavelength of NR12S depends on the fluidity of its membrane environment. Therefore, the NR12S emission can be used to study membrane fluidity. Liposomes doped with NR12S are excellent samples to evaluate the robustness of single particle profiling since liposomes with different lipid compositions have known membrane fluidity, which can be used to evaluate the sensitivity of the technique. Moreover, while two emission windows are used for NR12S to calculate the membrane fluidity, only one excitation wavelength is used, which makes the acquisition robust and easy. For this reason, fluorescence intensity traces in two channels were collected where every peak stands for single liposomes passing through the observation volume (Figure 1b). To evaluate membrane fluidity of each liposome, the generalized polarization (GP, an empirical index for membrane order) value was calculated for every peak using the red and green shifted emission wavelengths (see Materials and Methods section in the Supporting Information for details), and the GP histograms for different lipid compositions were generated as shown in Figure 1c. Liposomes composed of pure 1,2-dioleoyl-*sn*-glycero-3-phosphocholine (DOPC) show the lowest mean GP, whereas those of 1-palmitoyl-2-oleoyl-*sn*-glycero-3-phosphocholine and cholesterol (POPC/Chol) are the highest. GP distributions are fitted with Gaussian distributions, indicating a single population for each sample. However, standard deviation (designated as “σ”, which is the full width at half maxima) for POPC/Chol is higher than for the other two, displaying higher heterogeneity of membrane fluidity within this sample. This is due to imperfect mixing of lipids in multicomponent systems;^14^ hence, σ in SPP measurements can be used to investigate the sample heterogeneity.^15^

Mean GP values follow the same trend as in our previous work using conventional SPP despite different absolute values.^8^ This occurs when ratiometric GP measurements are performed at different microscopes or, as in this case, on the same device but using imaging mode instead of photon-counting mode. Sensitivities of the detectors are different in different parts of the spectrum, so the absolute GP values might not be preserved. Furthermore, when we used alternative photomultiplier tubes as detectors for the same measurements (Figure S1), we obtained the same trend in GP values but different GP mean and σ results. As a result, it is important to use certain “GP standards” (for example, artificial liposomes composed of a simple one-component lipid mixture) in case data sets acquired on different devices will be compared.

SPP can be employed to explore the absolute fluorescence intensities of single particles. A useful application of this type of analysis is the possibility of evaluating the amount of cargo within a vehicle particle (as we previously showed for RNA-loaded LNPs^8^). We also previously demonstrated that SPP can measure binding of different nanobodies to different virus-like particles^8^ or even estimate the precise number of antibodies bound to a single EV.^16^

To test the performance of Spot iSPP for such molecular quantification, we studied the impact of cholesterol on lipid–protein interactions, namely, on binding of carbohydrate-binding proteins (lectins) to their glycosphingolipid receptors. We chose two toxins of the AB_5_ family: Cholera toxin (Ctx) from *V. cholerae* and Shiga toxin (Stx) from *S. Dysenteriae*. Both toxins act similarly: they are taken up by cells and follow the retrograde pathway to the trans-Golgi network to block the biosynthesis of the proteins in the endoplasmic reticulum. Their cellular uptake is initiated at the plasma membrane by interaction of their B-subunits with corresponding glycosphingolipids (GSLs): globotriaosylceramide (Gb3) for StxB and monosialotetrahexosylganglioside (GM1) for CtxB. Lipid bilayer composition modulates the exposure of carbohydrate moieties to the extracellular space and, as a result, alters the binding efficiency of toxins to their corresponding receptors. The studies of the exact role of cholesterol in toxin–receptor interactions were performed in live cells by synthetic reconstitution and molecular dynamic simulations. Lingwood et al.^17^ demonstrated that binding of both StxB and CtxB was hindered by cholesterol in erythrocyte plasma membranes as well as in POPC/cholesterol membranes *in silico*. On the other hand, by reconstituting StxB–Gb3 binding in synthetic membranes, Schubert et al.^18^ showed that inclusion of cholesterol in DOPC liposomes promotes StxB binding to Gb3 receptor.

To address this controversy, we reconstituted binding of toxins to their receptors in synthetic liposomes composed of DOPC or POPC and 5 mol % of the respective GSL with 0, 15, or 30 mol % of cholesterol (Figure 2a). Liposomes were further supplemented with 0.01 mol % of Fast DiO fluorescent probe to label the membrane, while the toxins were labeled with Alexa 647. The traces recorded by Spot iSPP in lipid (Fast DiO) and protein (Alexa 647) channels are shown in Figure 2b. We detected liposomes in the lipid channel and quantified the signal in the protein channel for every liposome. The fluorescence signals showed a striking effect of cholesterol in protein binding (Figure 2). To convert fluorescence signal from proteins into the number of proteins per liposome, molecular brightness for both StxB-Alexa 647 and CtxB-Alexa 647 were calculated by measuring the fluctuations of both proteins in solution (Figure S2).

**Figure 2:**
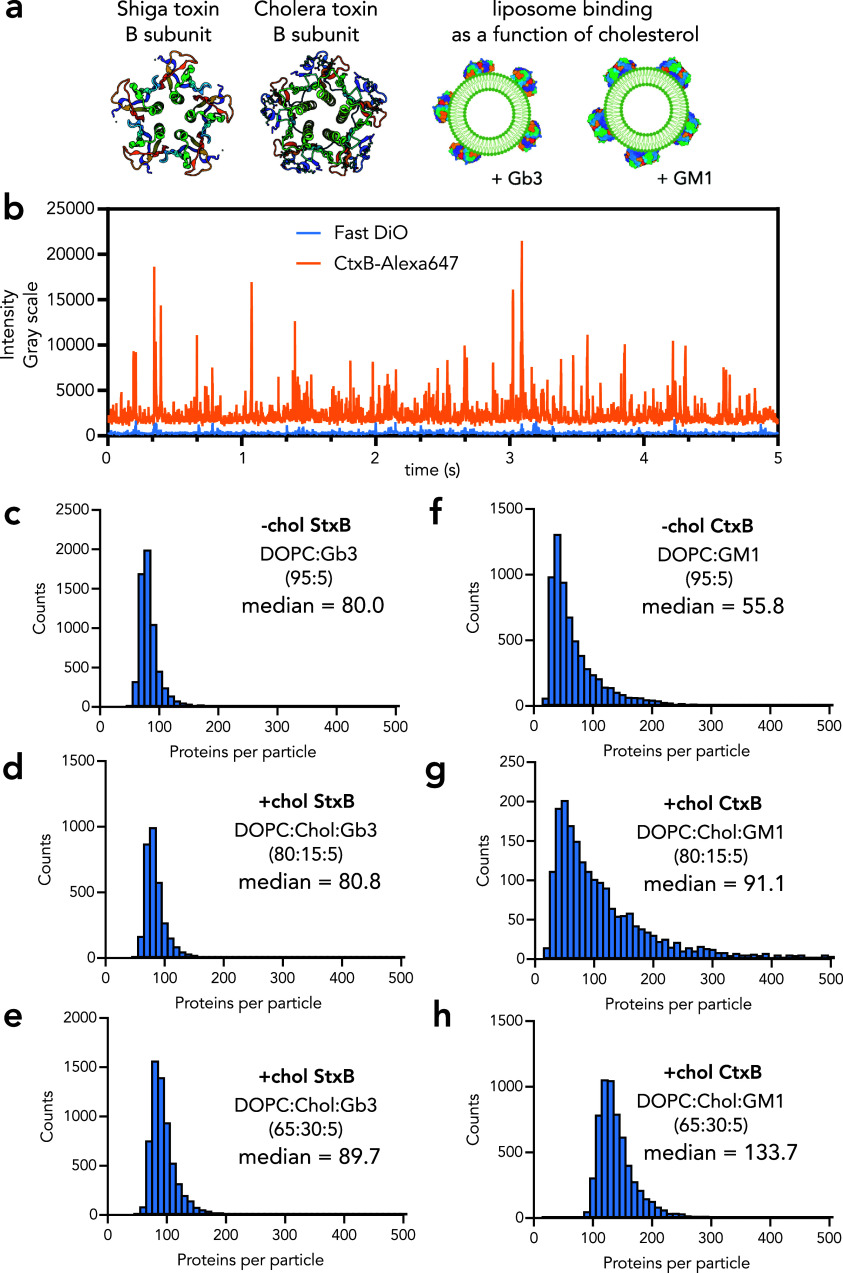
Studying lipid–protein interactions with spot iSPP. (a) Synthetic liposomes were prepared using the lipid mixtures of DOPC or POPC and cholesterol (0, 15 or 30 mol %) with 5 mol % of glycosphingolipids (Gb3 or GM1). StxB and CtxB were labeled by Alexa 647 and liposomes containing Fast DiO. (b) Intensity traces in Fast DiO and Alexa 647 channels for CtxB binding to liposomes containing DOPC/GM1 (95/5). (c–e) Protein per liposome analysis for StxB binding to the liposomes containing DOPC/Gb3 without and with cholesterol (15 and 30 mol %), respectively. The histogram is shifted to the right when cholesterol is present, which indicates an increase of StxB per liposome. (f–h) Protein per liposome analysis for CtxB binding to the liposomes containing DOPC/GM1 without and with (15 and 30 mol %) cholesterol, respectively. The histogram is shifted to the right when cholesterol is present which indicates an increase in CtxB per liposome. Data shown are representative of three independent measurements.

The histograms of the numbers of proteins per liposome are presented in Figure 2c–h. It is evident that, for both CtxB-GM1 and StxB-Gb3 interactions, an increase in the cholesterol amount in the membrane gradually improved the binding of the protein to its receptor. Moreover, the same binding improvement with addition of cholesterol was observed for the membranes composed of POPC instead of DOPC (Figure S2). By analyzing the co-occurrence of lipid and protein signals, we also quantified the percentage of liposomes bound by proteins for every condition (Table S1). For StxB-Gb3, the percentage of StxB positive liposomes increases with an increase of the cholesterol amount in the lipid bilayer. For CtxB-GM1, a similar trend was observed in DOPC but not in POPC vesicles. In POPC vesicles, the fraction of bound liposomes decreases in 15% Chol but increases in 30% cholesterol liposomes (Table S1).

These results show that both the phospholipid type and cholesterol content play a role in binding. The hindering effect of cholesterol on CtxB binding has previously been shown in POPC vesicles, but here, we show that the cholesterol amount in the lipid membrane also matters for binding.^17^ For StxB, cholesterol showed the improvement of StxB binding, which is in line with Schubert et al.^18^ but contradicts Lingwood et al.^17^

Spot iSPP can profile several thousand particles in a few minutes. Moreover, within short timeframes (≈2 min), it is possible to profile enough particles to resolve multiple subpopulations within the data distribution. This opens the possibility to perform several consecutive short-time acquisitions and as a result to explore the dynamic processes such as drug interaction with lipid bilayers. One widely used drug for manipulation of membrane composition is methyl-ß-cyclodextrin (MBCD). Cholesterol encapsulation by MBCD is widely used to remove cholesterol from native and artificial lipid bilayers, which is a standard procedure when the role of cholesterol in various biological processes is in question.^17,19−21^ Interestingly, it is also possible to use MBCD as an intermediary to deliver and distribute cholesterol homogeneously among artificial liposomes.^15^ MBCD interaction with membranes is complex especially when there are multiple different lipid environments present. To test which membrane environments MBCD preferentially interacts with, we prepared synthetic liposomes (Figure 3a) of POPC/Chol (70/30) and DPPC/Chol (70/30) and labeled them using NR12S. The mixture of these liposomes at 1:1 ratio was profiled and resulted in the GP histogram displayed in Figure 3b. This histogram consists of two distinct populations: the first population centered at −0.57 represents POPC/Chol liposomes and the second population at 0.45 represents DPPC/Chol liposomes. When MBCD (2 mM) is applied to POPC/Chol or DPPC/Chol separately, in both cases, it extracts some cholesterol, and the GP distribution shifts toward smaller GP values (Figure S3). MBCD also extracts some NR12S, but it is unlikely that signal from NR12S extracted by MBCD can contaminate results because the GP of NR12S incorporated in MBCD is extremely low (around −0.9, Figure S3e) and such population, if significant, would be clearly visible as a separate peak.

**Figure 3:**
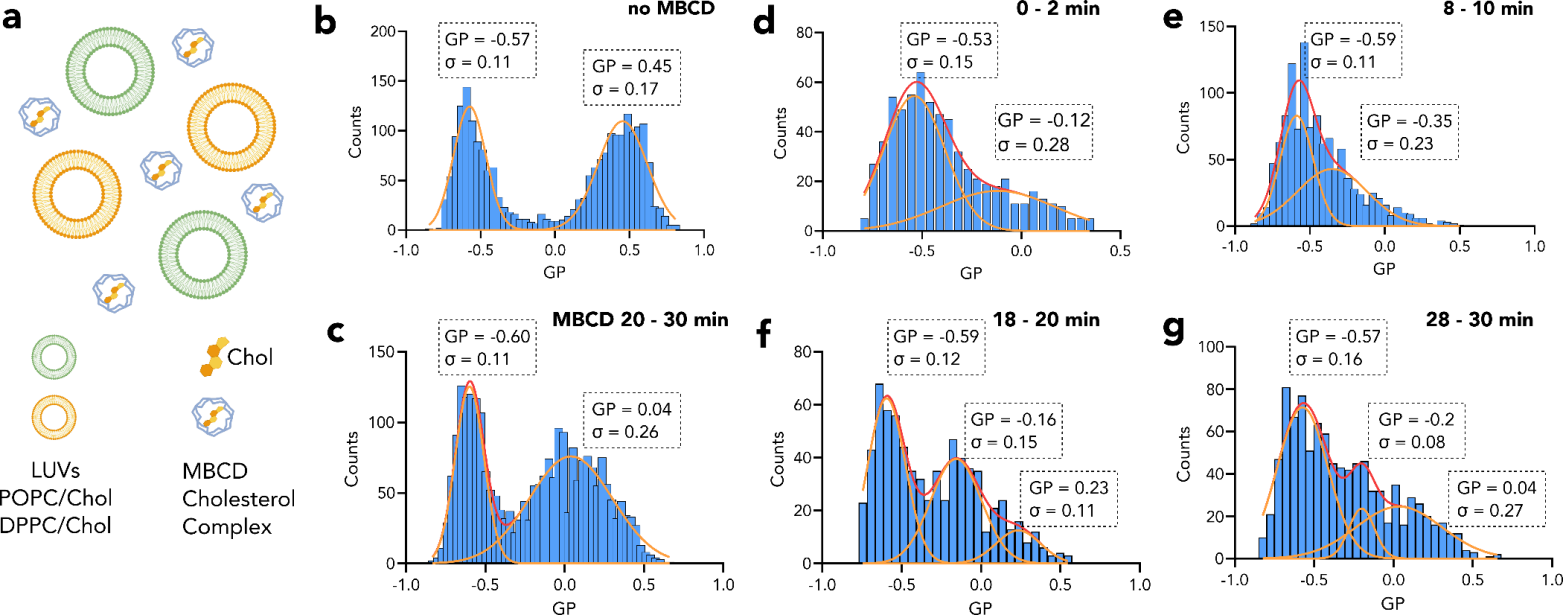
Cholesterol extraction from liposomes by MBCD. (a) Liposomes of POPC/Chol (70/30) and DPPC/Chol (70/30) were labeled with the environmentally sensitive probe NR12S and mixed. (b) GP histogram of POPC/Chol and DPPC/Chol liposomes mixed in the well plate at a ratio of 1:1. (c) GP histogram of POPC/Chol and DPPC/Chol mixture recorded between 20 and 30 min after MBCD application. (d–g) GP histogram of POPC/Chol and DPPC/Chol mixture recorded between (d) 0–2 min, (e) 8–10 min, (f) 18–20 min, and (g) 28–30 min after MBCD application. Data shown is representative of three independent measurements.

Interestingly, when MBCD (2 mM, 30 min) is applied to the mixture of POPC/Chol and DPPC/Chol, the population of POPC/Chol remains unchanged, whereas DPPC/Chol population shifts toward smaller GP values significantly (Figure 3c). It seems that MBCD prefers to extract cholesterol from DPPC/Chol liposomes rather than from POPC/Chol liposomes. Shaw et al.^22^ studied a similar problem by assessment of the chemical potential differences between POPC/Chol and DPPC/Chol at different cholesterol contents. According to their study, however, the chemical potential of POPC/Chol was higher compared to the chemical potential of DPPC/Chol at a cholesterol concentration of 30 mol %. This means that MBCD would more likely extract cholesterol from POPC/Chol first. DPPC/Chol has a higher chemical potential than POPC/Chol at a cholesterol concentration of at least 40–50 mol %. This necessitates time-lapse measurements to observe the time evolution of cholesterol extraction from different populations. Since we have access to various time points after MBCD application to the liposome mixture, we have insights on the dynamics of cholesterol extraction by MBCD. For this, we have recorded the intensity traces for 30 min continuously after 2 mM MBCD application to the liposome mixture, and its GP histogram before MBCD application is displayed in Figure 3b. After the acquisition was completed, the GP histograms were constructed for the shorter time traces of 2 min. These histograms represent transient states appearing and disappearing before they reach an equilibrium. Immediately after MBCD application (0–2 min), cholesterol is extracted from DPPC/Chol liposomes and the population of large GP values shifts left (Figure 3d). Moreover, at the 8–10 min time point, the high GP population is practically absent (Figure 3e). Later, however, the higher GP population reappears. At 18–20 min (Figure 3f) and at 28–30 min (Figure 3g) time points, there are more than two populations: lowest GP population still aligns well with POPC/Chol (GP ≈ −0.57), whereas the other two populations indicate large heterogeneity. Again, the traces acquired between 20 and 30 min (Figure 3c) have quite broad GP distribution. This population can consist of multiple minor subpopulations that are better pronounced in the transient histograms in Figure 3f,g. Of note, the POPC/Chol population changes little to none during 30 min after MBCD application. This is surprising as in Figure S3 it is demonstrated that cholesterol is removed from POPC/Chol liposomes when no DPPC/Chol is present in the solution. This data shows that drug interactions with membranes can be a complex phenomenon with multistep dynamics and intermediate steps. Specifically, the interaction of MBCD with membranes occurs as a function of the chemical potential of the membrane systems and MBCD itself. Therefore, during cholesterol removal by MBCD, there could be many intermediate chemical potential systems that play a role in drug action.

Biological nanosized particles (such as lipoproteins and extracellular vesicles) are widely used in basic and translational research and as biomarkers of diseases.^4,23^ Their small size and heterogeneity make it challenging to study them in a single particle and high throughput manner. Therefore, we aimed to use spot iSPP for studying nanoscale biological particles to profile their content and biophysical properties. To this end, we studied the membrane fluidity of EVs. EVs are small particles secreted by cells and used for cellular communication.^24,25^ Recently, they are also designed for therapeutic purposes.^16,26,27^ To be able to use them in biomedical research, it is crucial to shed light on their content heterogeneity. We extracted EVs from different cultured cells (Figure 4a–c) and labeled them with NR12S. The membrane fluidity analysis with iSPP resulted in one component GP distributions (Figure 4b) for all but with different mean fluidities. EVs isolated from HUVEC endothelial and HUAEC epithelial cell lines exhibited the most fluid membranes, whereas the GP of MSC-EVs was slightly higher. Fibroblast-derived EVs showed GP similar to that of T-lymphocytic EVs from Jurkat cells (the only suspension cell line in this collection), which were the least fluid EVs (Figure 4c). This shows that, depending on the cell source, the membrane content of the EVs varies dramatically. Moreover, biophysical heterogeneity should be considered for the efficacy of EV delivery and uptake by target cells. To show whether such heterogeneity exists in more physiologically occurring EVs, we used extracellular vesicles extracted from human donor urine (Figure 4d). EVs were isolated from three different donors and showed significant differences in their fluidity (Figure 4e,f). This shows that iSPP can be used to study properties and heterogeneity of nanoscale bioparticles from body fluids and can successfully show individual differences, which can be a new avenue for biomarker discovery.

**Figure 4:**
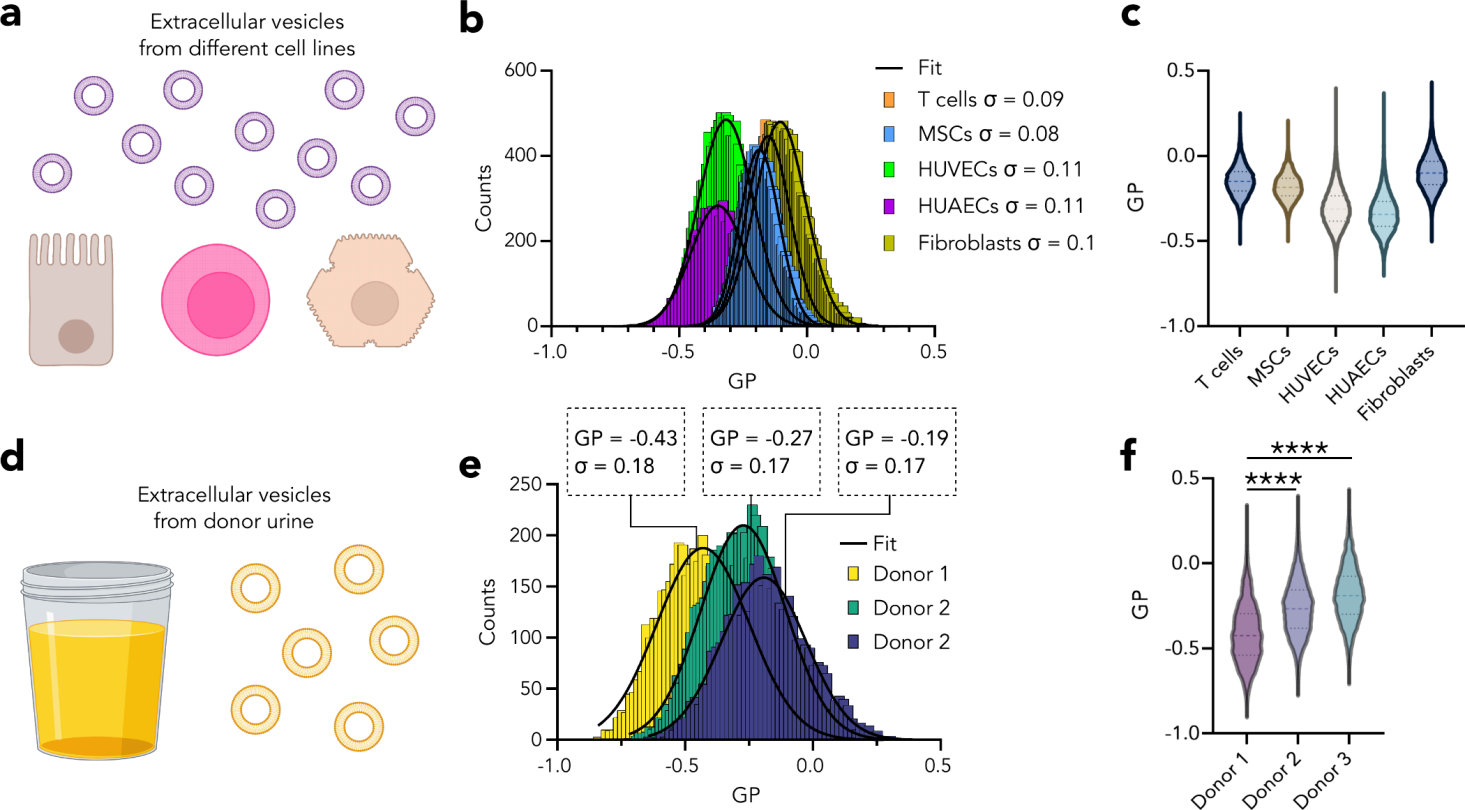
Membrane fluidity of the extracellular vesicles. (a) EVs were isolated from T cells, MSCs, HUVECs, HUAECs, and fibroblasts, labeled with the environmentally sensitive probe NR12S, and profiled using spot iSPP. (b) GP histograms of EVs isolated from various cells in a culture. (c) GP violin plot of EVs in (b). (d) EVs were isolated from urine of three donors, labeled with NR12S, and profiled using spot iSPP. (e) GP histograms of EVs isolated from donor urine. (f) GP violin plot of EVs in (e). Data shown is representative of three independent measurements.

Nanoscale bioparticles are important biomarkers for diseases and crucial tools for biomedicine. Therefore, their content, heterogeneity, and properties have significant implications for understanding physiology and diseases as well as using them as biomedical tools. Methodologies to study these particles are limited; however, there are several initiatives to overcome this technological bottleneck. SPP is one of these techniques that we developed recently to make single particle measurements possible on commercially available microscopes in a high throughput and single particle manner.

In this work, we develop a new version of SPP: Spot imaging SPP (iSPP), which is more accessible for users with less advanced microscopy experience as well as for users with limited access to advanced confocal microscopes equipped with photon counting detectors. Spot iSPP was validated here with artificial and native lipid particles for two-color profiling as well as for biophysical characterization. It is available for microscopes with imaging (integration) mode and will assist in single particle analysis of bioparticles in health and disease, for lipid and protein profiling, and for deciphering biophysical disease biomarkers.

## Data Availability

All data will be available via Figshare upon publication at the DOI: 10.17044/scilifelab.26048629.

## Supporting Information Available

Materials and methods and supplementary figures and tables, including additional methodology details (Figure S1), additional quantification of drug binding to liposomes (Figures S2–S3 and Table S1), and detailed exosome characterization (Figure S4) (PDF)
