# Supplementary material for: Imaging Single Particle Profiler to Study Nanoscale Bioparticles Using Conventional Confocal Microscopy
Source: Nano Lett. 2025 Jan 29;25(6):2173–80. doi: 10.1021/acs.nanolett.4c05117 (PMC11827106; doi:10.1021/acs.nanolett.4c05117)
Supplement: Supplementary file 1 — nl4c05117_si_001.pdf [file nl4c05117_si_001.pdf]

Supporting Information

Imaging single particle profiler to study nanoscale bioparticles using conventional confocal microscopy

Taras Sych [a,], André Görgens [b,c], Loïc Steiner [d,e], Gozde Gucluler [d,e], Ylva Huge [f], Farhood Alamdari [g], Markus Johansson [h], Firas Aljabery [i], Amir Sherif [j], Susanne Gabrielsson [d,e], Samir EL Andaloussi [b,c] and Erdinc Sezgin [a,*]*

[a] Science for Life Laboratory, Department of Women's and Children's Health, Karolinska Institutet, Tomtebodavägen 23, 17165 Solna, Sweden

[b] Division of Biomolecular and Cellular Medicine, Department of Laboratory Medicine, Karolinska ATMP Center, Karolinska Institutet, 14152 Huddinge, Sweden.

[c] Department of Cellular Therapy and Allogeneic Stem Cell Transplantation (CAST), Karolinska University Hospital, 14152 Huddinge, Sweden.

[d] Division of Immunology and Respiratory Medicine, Department of Medicine Solna, Karolinska Institutet, 17164 405 Stockholm, Sweden.

[e] Department of Clinical Immunology and Transfusion Medicine, Center for Molecular Medicine, Karolinska University Hospital, 17164 Stockholm, Sweden.

[f] Department of Urology in Östergötland, and Department of Biomedical and Clinical Sciences, Linköping University, 58225 Linköping, Sweden

[g] Department of Urology, Vastmanland Hospital, 72189 Västerås, Sweden

[h] Departement of Surgery and Urology, county hospital of Sundsvall-Härnösand, 85643 Sundsvall, Sweden.

[i] Department of Urology in Östergötland, and Department of Biomedical and Clinical Sciences, Linköping University, Linköping, Sweden

[j] Department of Diagnostics and Intervention, Umeå university, 90187 Umeå, Sweden

E-mail: erdinc.sezgin@ki.se and taras.sych@ki.se

Materials and Methods

Preparation of LUVs.

1,2-Dioleoyl-sn-glycero-3-phosphocholine (DOPC), 1-palmitoyl-2-oleoyl-sn-glycero-3-phosphocholine (POPC), Dipalmitoylphosphatidylcholine (DPPC) and cholesterol (Chol) were from Avanti polar lipids. Glycosphingolipid globtriaosylceramide (Gb3) was from Larodan, monosialotetrahexosylganglioside was from Sigma. Fluorescent lipids and lipid-like probes we used: FastDiO (ThermoFisher), NR12S (Bio-Techne). Lipid mixtures in chloroform at 0.25 mg/ml were prepared: pure DOPC, pure POPC, POPC/ Chol (70/30), DPPC/ Chol (70/30). DOPC/Chol/Gb3 (65/30/5), DOPC/Chol/Gb3 (80/15/5), DOPC/Gb3 (95/5), DOPC/Chol/GM1 (65/30/5), DOPC/Chol/GM1 (80/15/5), DOPC/GM1 (95/5), POPC/Chol/Gb3 (65/30/5), POPC/Chol/Gb3 (80/15/5), POPC/Gb3 (95/5), POPC/Chol/GM1 (65/30/5), POPC/Chol/GM1 (80/15/5), POPC/GM1 (95/5). Mixture including GM1 or Gb3 were further supplemented with 0.01 mol % of FastDIO. Mixtures were dried under the flow of nitrogen, rehydrated with buffer (150 mM NaCl, 10 mM Hepes, 2 mM $CaCl_2$) and vortexed harshly to form multilamellar vesicles. Then, the suspension of MLVs was sonicated at power 3, duty cycle 40% for 10 mins using Branson Sonifier 250. LUVs were stored at 4 degrees under nitrogen. For membrane fluidity studies, the solutions of LUVs (0.25 mg/ml) were incubated with 1 µM Nile Red 12 S (NR12S, stock concentration of 10 µM in DMSO) directly before profiling.

Lectin labelling and application

The pentameric B-subunit of Shiga toxin (StxB) and the pentameric B-subunit of Cholera toxin were purchased from Sigma Aldrich. Lectins were labeled with Alexa-647 (NHS ester) from Thermofisher. Briefly, 1 µL of a 10 mg/mL solution of the amino-reactive probe dissolved in DMSO was added to 100 µL of a 0.5 mg/mL protein solution in PBS (-/-, Gibco) supplemented with 100 µM NaHCO3, pH 8.5. The mixture was incubated for 1 h at room temperature under continuous shaking. The labeled lectins were purified using Zeba Spin Desalting Columns (0.5 mL, MW cut-off: 7.0 kDa) from ThermoFisher Scientific.

For measurements of StxB and CtxB in solution they were brought to the concentration of 100 nM in PBS. For binding studies 10 µl of LUVs that contained appropriate GSL

receptor were incubated with 1 μM of lectin for 15 minutes before profiling with Spot SPP.

MBCD application

Methyl-ß-cyclodextrin (MBCD) was from Sigma. MBCD was freshly dissolved with ultrapure water at a concentration of 40 mM. For control experiments, LUVs (POPC/Chol or DPPC/Chol) were incubated for 30 min with 2 mM of MBCD and diluted 10 times for profiling with Spot SPP. For control experiment on fluorescent dye capture by MBCD, 2 mM of MBCD was mixed with 1 μM solution of NR12S, incubated for 30 min and diluted 10 times for Spot SPP profiling. For the cholesterol extraction dynamics study, POPC/Chol and DPPC/Chol (1:1) mixture was added to the profiling well plate and the initial 10 min measurement was performed. Later, 2 mM of MBCD was added to exactly the same well, and Spot-SPP profiling was started immediately after MBCD addition. Traces were recorded for 30 minutes and later split into shorter traces in order to analyze the time series.

Cell culture and isolation of extracellular vesicles.

EVs were prepared from Jurkat T-lymphocyic suspension cells, CB-MSCs, HUVEC, HUAEC, and fibroblasts. Cell lines were cultured in the following media: Jurkat T cells were cultured in RPMI1640 medium. CBMSCs (MSCs; ATCC, PCS-500-010, Umbilical Cord-Derived Mesenchymal Stem Cells, Normal, Human) were cultured in MEM-α modification medium (containing L-glutamine; ThermoFisher Scientific) supplemented with 5 ng/ml of bFGF (Sigma, F0291). Immortalized human umbilical vein endothelial cells (HUVEC/TERT2; ATCC #CRL-4053) were cultured in Vascular Cell Basal Medium (ATCC #PCS-100-030) supplemented with endothelial cell growth Kit-VEGF (ATCC #PCS-110-041). HUAEC (CI-huAEC; Inscreenex, human airway epithelial cells) were cultured in huAEC media (INS-ME-1013-500ml; Inscreenex) supplemented with 1% sodium pyruvate (ThermoFisher). BJ-5ta fibroblast cells (ATCC CRL-4001) were cultured with 4:1 mixture of Dulbecco's modified Eagle's medium (containing 4 mM L-glutamine, 4.5 g/L glucose and 1.5 g/L sodium bicarbonate) and Medium 199 (0.01 mg/ml Hygromycin B/10687010, Thermo Fisher) supplemented with 10% FBS (ThermoFisher). Unless indicated otherwise, all cells were supplemented with 10% FBS (Invitrogen) and 1X Antibiotic-Antimycotic (Anti-Anti) (ThermoFisher Scientific).

All cell lines were grown at 37°C, 5% CO_2 in a humidified atmosphere and regularly tested for the presence of mycoplasma. For EV harvesting, cell culture-derived conditioned media (CM) was changed to OptiMem (Invitrogen) 48 h before harvest of conditioned media as described before[1]. Unless indicated otherwise, all conditioned media samples were directly subjected to a low-speed centrifugation step at 500 xg for 5 min followed by a 2,000 xg spin for 10 min to remove larger particles and cell debris. Pre-cleared cell culture supernatant was subsequently filtered through 0.22 µm bottle top vacuum filters (Corning, cellulose acetate, low protein binding) to remove any larger particles. EVs were first prepared by tangential flow filtration (TFF) by using a KR2i TFF system (SpectrumLabs) equipped with modified polyethersulfone (mPES) hollow fiber filters with 300 kDa membrane pore size (MidiKros, 370 cm2 surface area, SpectrumLabs) at a flow rate of 100 mL/min (transmembrane pressure at 3.0 psi and shear rate at 3700 sec−1) as described previously [2]. Amicon Ultra-0.5 10 kDa MWCO spin-filters (Millipore) were used to concentrate the sample to a final volume of 100 µL. The sample was then loaded on a qEV column (Izon Science) and the EV fractions were collected according to the manufacturer's instructions. Final EV samples were stored at −80°C in PBS-HAT [PBS supplemented with HEPES, human serum albumin and D-(+)-Trehalose dihydrate] buffer until usage[3]. Before studying with SPP, extracellular vesicles were unfrozen at room temperature and incubated with 5 µM of NR12S for 10 minutes. 40 traces 15 seconds each were recorded for every sample. Additionally, the size of EVs was assessed by dynamic light scattering (DLS, Figure S4a) using Malvern Zetasizer.

Urine collection and extracellular vesicles isolation

Three patients with non-muscle invasive urothelial urinary bladder cancer, staged TaG1, TaG2 and T1G3 respectively, were prospectively recruited between 2016 and 2018 and urine was obtained prior to the primary transurethral resection of the bladder tumor (TURBT). The three patients were 69, 61 and 78 years of age: two females and one male. All samples were shipped and processed fresh the day of surgery. All experimental protocols were approved by the Regional Ethical Review Board in Stockholm (original no.: 2007/71- 31), and all patients were above 18 and gave written and oral informed consent. Urine samples were sequentially centrifuged at 300 xg for 10 min, 3000 xg for 30 min, 10,000 xg (Ti45 rotor, Beckman Coulter, tube average) for

30 min and filtered through a 0.22µm filter. The urine was then up concentrated by tangential flow filtration (100kDa cut-off, VivaFlow). After storage at -80ºC, extracellular vesicles were purified by ultracentrifugation at 100,000 xg (Ti45 rotor, Beckman Coulter, tube average) for 2h, and the pellet was resuspended in a small volume of PBS and stored at -80ºC until further analysis. Before studying with SPP, extracellular vesicles were unfrozen at room temperature and incubated with 5 µM of NR12S for 10 minutes. 40 traces 15 seconds each were recorded for every sample. Additionally, the size of EVs was assessed by dynamic light scattering (DLS, Figure S4b) using Malvern Zetasizer.

Single particle profiling measurements and analysis.

Single Particle Profiling was performed using the setup for confocal acquisition („spot scan") at a Zeiss LSM 780 microscope. A 488 nm argon ion laser was used for FastDiO and NR12S excitation, whereas a 633 nm He−Ne laser was used for Alexa 647. A 40×1.2 NA water immersion objective was used to focus the light. The laser power was set to 1% of the total laser power that corresponds to 20 µW. The fluorescence emission was detected by GaAsP spectral detector in integration mode, with HV Gain of 900 – 1100.The emission detection windows were set as 490 – 560 for FastDiO and 650 – 700 for Alexa647. Emission from NR12S was recorded simultaneously in both channels. Intensity traces were recorded between10 and 30 minutes and later split if required. The detailed description and guide on data acquisition is available as video tutorial here:
https://www.youtube.com/watch?v=IicPvjPySDY&list=PLvnnxg3kwLpWX5M-e-14hhWQVPsPEQ5jB.

Traces and curves were then analysed using "Py Profiler", our in-house python program using the python packages: tkinter (v. 8.6.10), matplotlib (v. 3.3.4), lmfit (v. 1.0.2), ttkwidgets (v. 0.10.0), scipy (v. 1.6.2), seaborn (v. 0.11.1), pandas(v. 1.2.4). The source code as well as the standalone distributions for Windows and Mac are available at the Github: https://github.com/taras-sych/Single-particle-profiler/tree/Release-v-3.0.

Peak analysis. Briefly, individual peaks from the traces were identified and intensities for these individual peaks were extracted with further calculation of generalized polarization (GP) if applicable. GP was calculated using the formula:

$$GP = \frac{I_{blue} - I_{red}}{I_{blue} + I_{red}};$$

where I_{blue} – fluorescence intensity in short wavelength („blue“) region of the emission spectrum and I_{red} – fluorescence intensity in long wavelength („red“) region of the emission spectrum.

The description and guide to the program is available as a video tutorial. The link to the video is at https://www.youtube.com/watch?v=p5afSZSkbfE.

Diffusion analysis was also performed for quantification of brightness of proteins in solution using Py Profiler. Curves were fitted with the following three-dimensional diffusion:

$$G(\tau) = \frac{1}{N}\left(1 + \frac{\tau}{\tau_D}\right)^{-1}\left(1 + \frac{\tau}{AR^2\tau_D}\right)^{-\frac{1}{2}}$$

where *N* represents the number of fluorescent species within the beam’s focal volume. Next, molecular brigtness was quantified by dividing the mean value of the trace intensity by *N*.

Statistical analysis

Where applicable, the non-parametric Kruskal-Wallis ANOVA was used for determining the statistical significance. P-values are indicated in the figure legends.

References

(1) Hagey, D. W.; Ojansivu, M.; Bostancioglu, B. R.; Saher, O.; Bost, J. P.; Gustafsson, M. O.; Gramignoli, R.; Svahn, M.; Gupta, D.; Stevens, M. M.; Görgens, A.; El Andaloussi, S. The Cellular Response to Extracellular Vesicles Is Dependent on Their Cell Source and Dose. *Sci. Adv.* **2023**, *9* (35), eadh1168. https://doi.org/10.1126/sciadv.adh1168.

(2) Corso, G.; Mäger, I.; Lee, Y.; Görgens, A.; Bultema, J.; Giebel, B.; Wood, M. J. A.; Nordin, J. Z.; Andaloussi, S. E. Reproducible and Scalable Purification of Extracellular Vesicles Using Combined Bind-Elute and Size Exclusion Chromatography. *Sci. Rep.* **2017**, *7* (1), 11561. https://doi.org/10.1038/s41598-017-10646-x.

(3) Görgens, A.; Corso, G.; Hagey, D. W.; Jawad Wiklander, R.; Gustafsson, M. O.; Felldin, U.; Lee, Y.; Bostancioglu, R. B.; Sork, H.; Liang, X.; Zheng, W.; Mohammad, D. K.; van de Wakker, S. I.; Vader, P.; Zickler, A. M.; Mamand, D. R.; Ma, L.; Holme, M. N.; Stevens, M. M.; Wiklander, O. P. B.; El Andaloussi, S. Identification of Storage Conditions Stabilizing Extracellular Vesicles Preparations. *J. Extracell. Vesicles* **2022**, *11* (6), e12238. https://doi.org/10.1002/jev2.12238.

Supplementary Figures

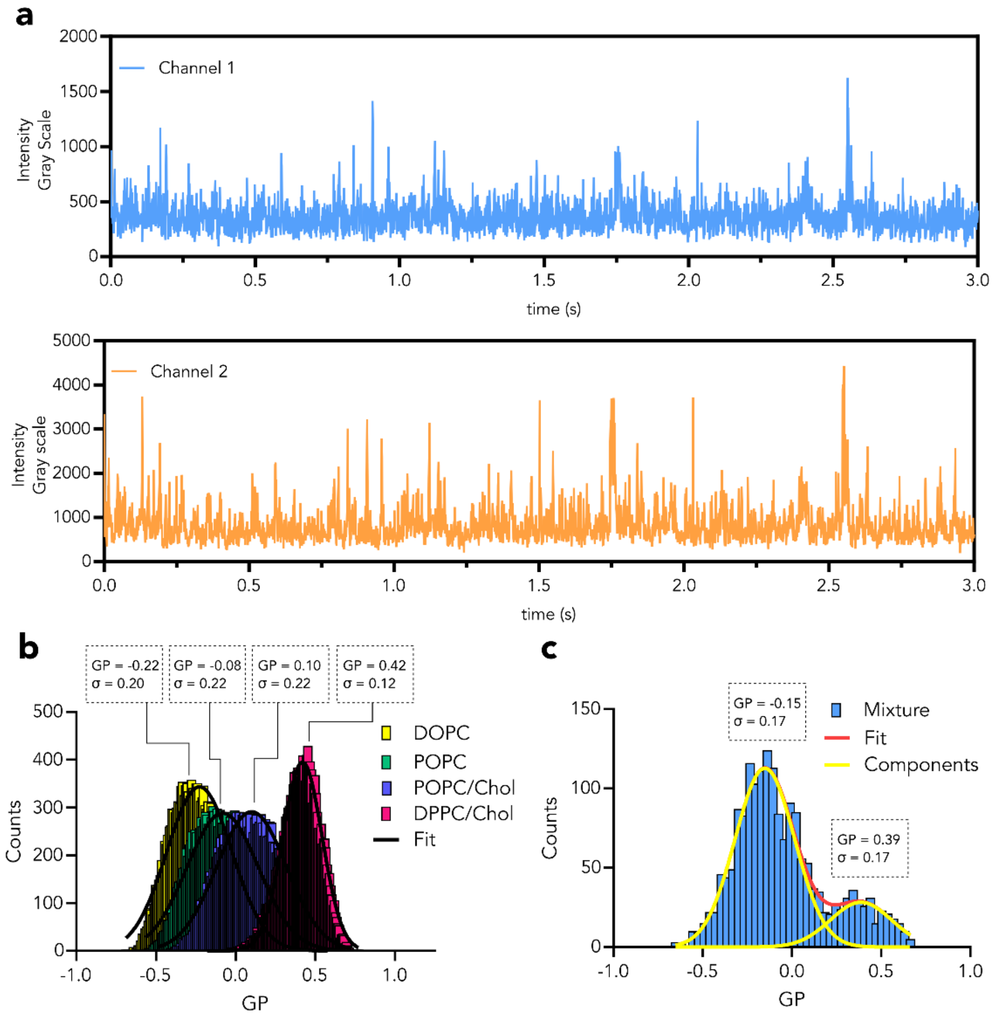

Figure S1. Spot-SPP performed with PMT detectors. **a)** The objective is fixed on a spot and PMT detectors in integration mode record the fluorescence signal over time in multiple channels in imaging mode. Particles are diffusing through the observation spot hence the signal provides intensity fluctuations over time. Fluorescence fluctuations recorded over time in two channels: green channel (490 – 561 nm) and red channel (650 – 700 nm). Synthetic liposomes with distinct lipid compositions (pure DOPC, pure POPC and POPC/chol (70/30)) are labeled with environment sensitive probe NR12S and fluorescence intensities for single particles in “blue-shifted” and “red-shifted” channels were recorded; **b)** The resulting generalized polarization (GP) histograms for different lipid compositions; **c)** The GP histogram obtained from mixture of all liposomes presented in **b**.

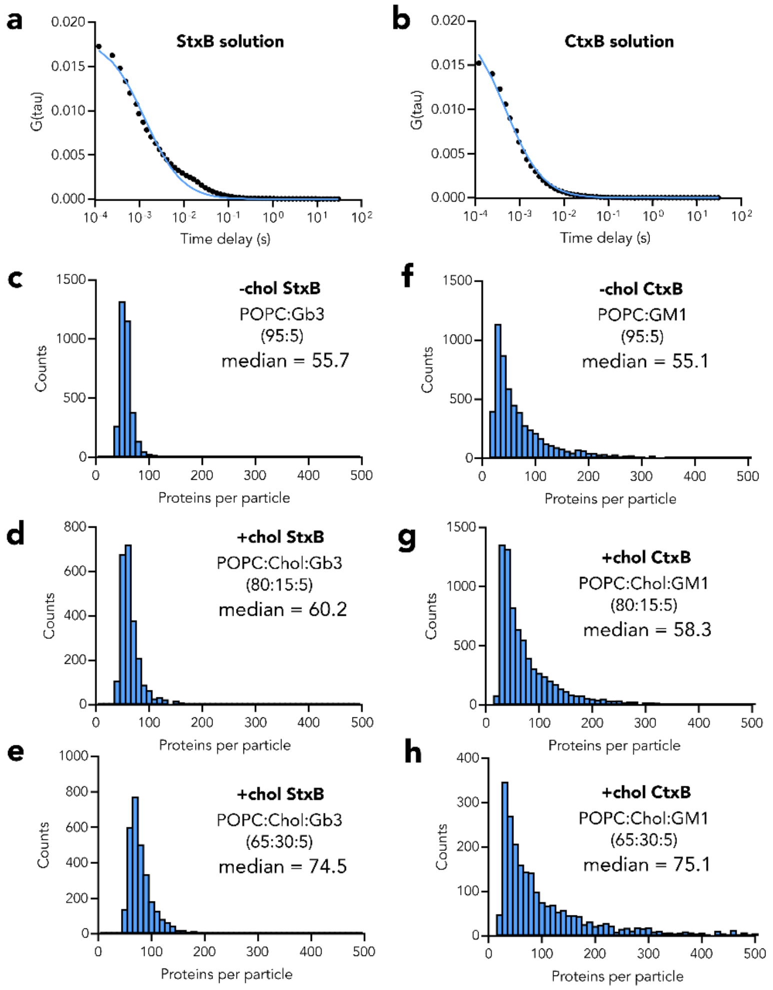

Figure S2. Lectin binding to LUVs composed of POPC and cholesterol that contain glycosphingolipid receptors. a) Autocorrelation function and its fit obtained from fluorescence fluctuations from StxB in solution; b) Autocorrelation function ant its fit obtained from fluorescence fluctuations from CtxB in solution; c-e) histograms of StxB binding to LUVs composed of POPC and cholesterol; f-h) histograms of CtxB binding to LUVs composed of POPC and cholesterol.

Table S1: Number of liposomes bound by lectins

		Cholesterol	Total peaks	Lectin positive peaks	Lectin positive peaks percentage
StxB Gb3	POPC	0%	6466	645	9.98 %
		15%	4478	863	19.27 %
		30%	5586	1720	30.79 %
	DOPC	0%	6235	1044	16.74 %
		15%	3351	776	23.16 %
		30%	6296	1887	29.97 %
CtxB GM1	POPC	0%	4071	2766	67.94 %
		15%	3681	2253	61.21 %
		30%	917	654	71.32 %
	DOPC	0%	3117	1706	54.73 %
		15%	3681	2253	61.21 %
		30%	917	754	82.22 %

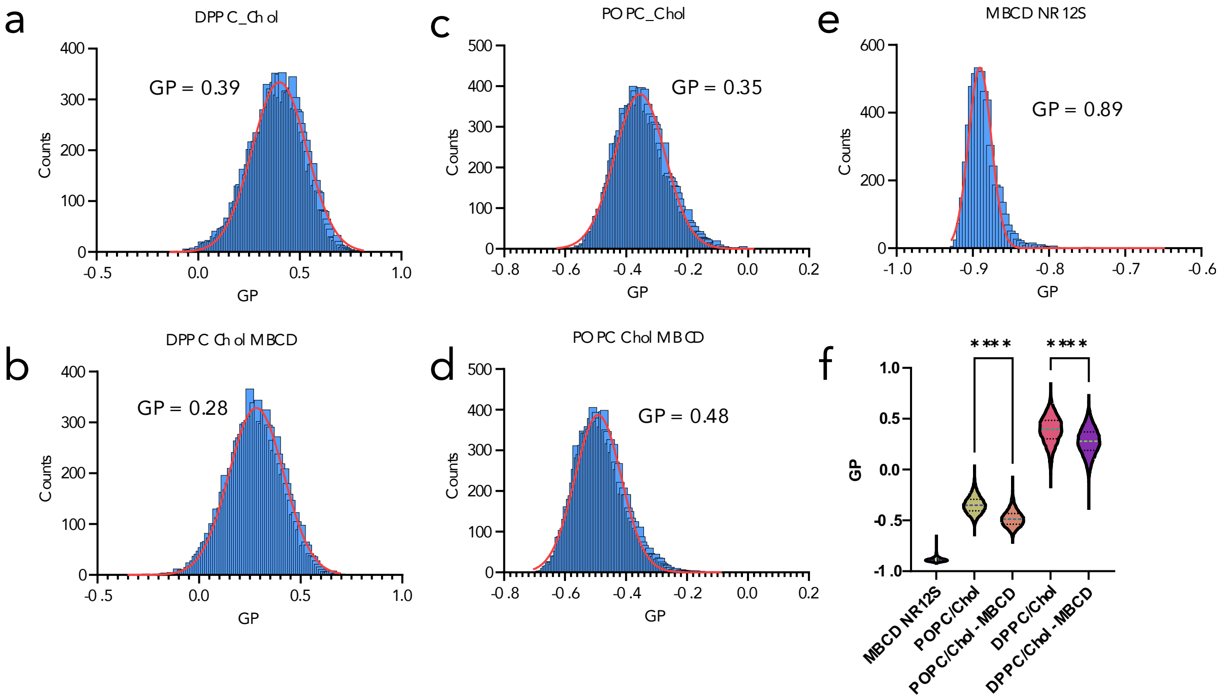

Figure S3. MBCD impact on LUVs. a) GP histogram of DPPC/Chol LUVs before 2 mM MBCD application; b) GP histogram of DPPC/Chol LUVs after 2 mM MBCD application; c) GP histogram of POPC/Chol LUVs before 2 mM MBCD application; d) GP histogram of POPC/Chol LUVs after 2 mM MBCD application; e) GP histogram of the solution of NR12S incubated with MBCD; f) Violin plots of GP for a-e, **** stands for *p<0.0001.*

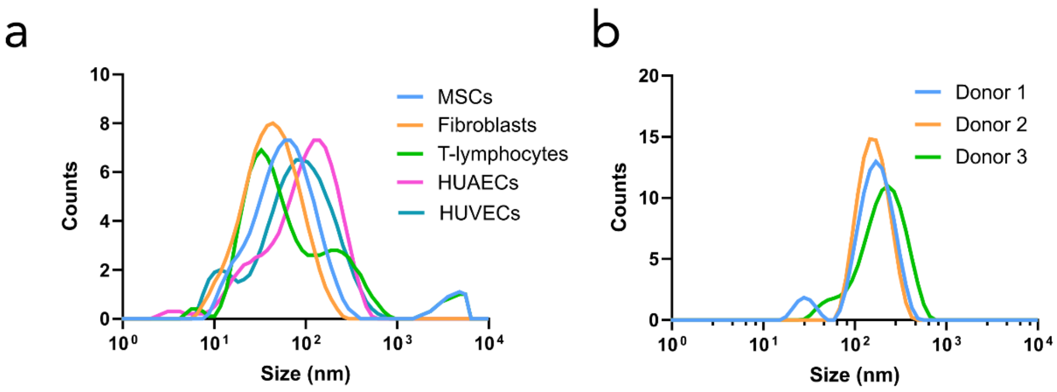

Figure S4. Extracellular vesicle size studied with DLS; a) EVs extracted from cells; b) EVs isolated from patient urine.
